# Simulated Microgravity Suppresses Osteogenic Differentiation of Mesenchymal Stem Cells by Inhibiting Oxidative Phosphorylation

**DOI:** 10.3390/ijms21249747

**Published:** 2020-12-21

**Authors:** Lin Liu, Yansiwei Cheng, Jie Wang, Zhongjie Ding, Alexander Halim, Qing Luo, Guanbin Song

**Affiliations:** Key Laboratory of Biorheological Science and Technology, Ministry of Education, College of Bioengineering, Chongqing University, Chongqing 400030, China; liulin@cqu.edu.cn (L.L.); chengyansiwei@cqu.edu.cn (Y.C.); wangjie518@cqu.edu.cn (J.W.); dingzj@cqu.edu.cn (Z.D.); 20161500175@cqu.edu.cn (A.H.); qing.luo@cqu.edu.cn (Q.L.)

**Keywords:** simulated microgravity, mesenchymal stem cells, osteogenesis, oxidative phosphorylation, Sirt1

## Abstract

Studies showed that energy metabolism plays a pivotal role in the differentiation of stem cells. Previous studies revealed that simulated microgravity (SMG) inhibits osteogenic differentiation of mesenchymal stem cells (MSCs). However, the underlying relationship between osteogenesis and energy metabolism under SMG conditions is not fully understood. In the present study, we investigated mitochondrial oxidative phosphorylation (OXPHOS) by assessing the level of peroxisome proliferator activated receptor γ coactivator 1α (PGC-1α), mitochondrial DNA (mtDNA) copy number, mitochondrial mass and oxygen consumption rate (OCR) during osteogenesis of MSCs under SMG conditions. We found that SMG inhibited osteogenic differentiation and OXPHOS of MSCs. Moreover, the expression of sirtuin 1 (Sirt1), an important energy sensor, significantly decreased. After upregulating the expression of Sirt1 using resveratrol, an activator of Sirt1, SMG-inhibited OXPHOS and osteogenic differentiation of MSCs were recovered. Taken together, our results suggest that SMG suppresses osteogenic differentiation of MSCs by inhibiting OXPHOS, indicating that OXPHOS might serve as a potential therapeutic target for repairing bone loss under microgravity conditions.

## 1. Introduction

One of the health problems that astronauts face after a long spaceflight is bone loss. Mesenchymal stem cells (MSCs) are multipotent stromal cells that are mainly derived from bone marrow and are the major source for osteoblasts, which have become a perfect candidate for use in clinical application [1]. A growing body of evidence indicates that osteogenic differentiation of MSCs is evidently inhibited by weightlessness [2], which means that gravity is necessary and crucial for the regulation of bone remodeling and homeostasis [3]. In our previous study, we showed that simulated gravity (SMG) could inhibit osteogenic differentiation of MSCs [4]. Taken together, bone loss induced by SMG is very likely due to the reduced osteogenesis of MSCs.

The state of energy metabolism is able to affect the differentiation of MSCs. Energy metabolism can shift from glycolysis to mitochondrial oxidative phosphorylation (OXPHOS) during the osteogenic differentiation of MSCs in vitro [5]. In addition, studies confirmed that OXPHOS is important and necessary for osteogenic differentiation of MSCs in vitro [6,7], which indicates that OXPHOS plays a crucial role in the process of osteogenesis. However, to our knowledge, the role of OXPHOS in osteogenic processes of MSCs in microgravity is poorly explored. Thus, it is meaningful to explore the relationship between OXPHOS and osteogenic differentiation of MSCs under SMG conditions.

Sirtuin 1 (Sirt1) is a histone deacetylase dependent on nicotinamide adenine dinucleotide (NAD+), which is known as an energy sensor involved in cell metabolism and mitochondrial regulation [8,9]. Additionally, many studies recently showed that Sirt1 has a vital role in osteogenesis of MSCs, suggesting that Sirt1 can regulate osteogenesis of MSCs [10,11,12,13] and improve bone impairment [14]. Resveratrol, an activator of Sirt1, can increase the expression of Sirt1 to treat osteoarthritis [15]. Thus, Sirt1 has become a new target in bone metabolism in recent years. Furthermore, some results confirm that Sirt1 can regulate key molecules for mitochondrial biogenesis, such as peroxisome proliferator activated receptor γ coactivator 1α (PGC-1α), which is involved in OXPHOS [16]. Nevertheless, little is known about the role of Sirt1 in OXPHOS during the osteogenesis of MSCs in the conditions of SMG.

We therefore explored the role of energy metabolism in osteogenic differentiation of MSCs under SMG conditions, by investigating the changes in OXPHOS and Sirt1 levels in this study. Our data indicate that the inhibition of osteogenesis of MSCs induced by SMG is due to decreased Sirt1 expression, followed by the repression of OXPHOS.

## 2. Results

### 2.1. SMG Hampered Osteogenic Differentiation and OXPHOS in MSCs

**Figure 1:**
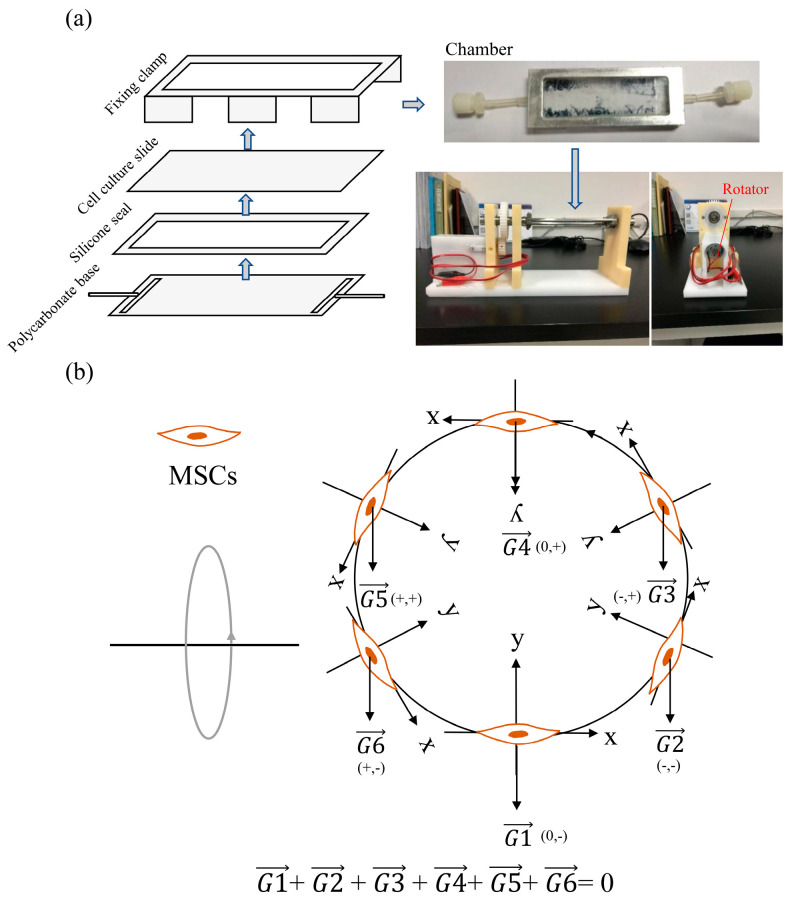
Simulated microgravity rotary flat chamber bioreactor. (**a**) Photograph of the clinostat used in this study to model simulated microgravity. (**b**) Schematic diagram explaining the gravity feeling of MSC on uniaxial clinostat used in this study. A system of rectangular coordinates based on the MSC is established. The gravity vector of MSCs changes constantly, following the rotating of the clinostat. The average gravity vector of MSCs is nulled after rotating one circle, as a result, the MSC could not feel the gravity.

**Figure 2:**
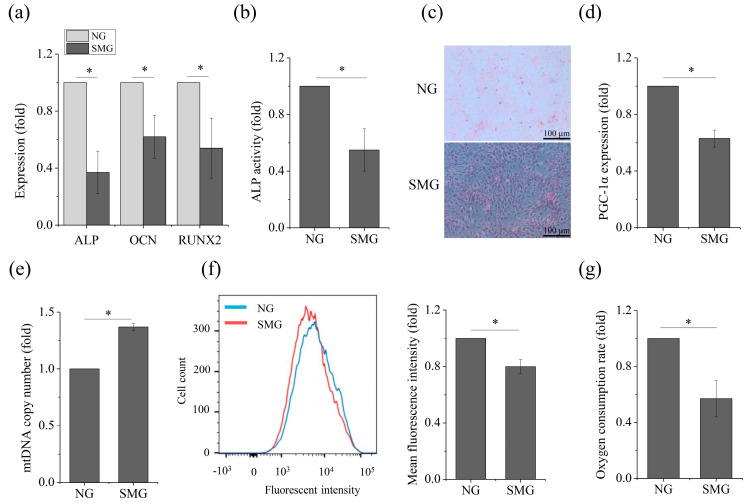
SMG inhibits osteogenesis and OXPHOS in MSCs after exposure to SMG. (**a**) Gene expression of ALP, OCN, RUNX2 after 72 h. (**b**) Relative ALP activity after 72 h. (**c**) Representative images of ALP staining after 7 day. (**d**) Gene expression of PGC-1α after 72 h. (**e**) Relative mtDNA copy number levels after 72 h. (**f**) Cells were stained with MitoTracker Green for mitochondrial mass and assessed by flow cytometry after 72 h. (**g**) Relative OCR levels after 72 h. For each group, the values are the mean ± SEM from three representative independent experiments. Control: NG control. * *p* < 0.05. NG, normal gravity; SMG, simulated microgravity. Scale bar: 100 μm.

A two-dimensional clinostat device presented in the Figure 1 was used to stimulate microgravity in this study. We first assessed the effect of SMG on osteogenic differentiation of MSCs by testing the mRNA expression levels of osteogenic markers such as ALP, OCN, and RUNX2 by qRT-PCR. Figure 2a shows that the expression of the osteogenic-related markers obviously decreased under SMG conditions, compared to that of NG. ALP activity also obviously decreased under SMG conditions (Figure 2b). Additionally, a significant reduction for positive ALP staining of cells was observed under SMG conditions (Figure 2c). Next, we explored the effect of SMG on OXPHOS, as osteogenesis of MSCs was impaired. Although we observed an increasing level of mtDNA copy number (Figure 2e), we saw a significant decrease under the SMG conditions, in the expression of PGC-1α (Figure 2d), the mitochondrial mass (Figure 2f), and OCR (Figure 2g). Taken together, these results demonstrated that SMG hampered both osteogenesis and OXPHOS in MSCs.

### 2.2. SMG Inhibited Sirt1 Expression

**Figure 3:**
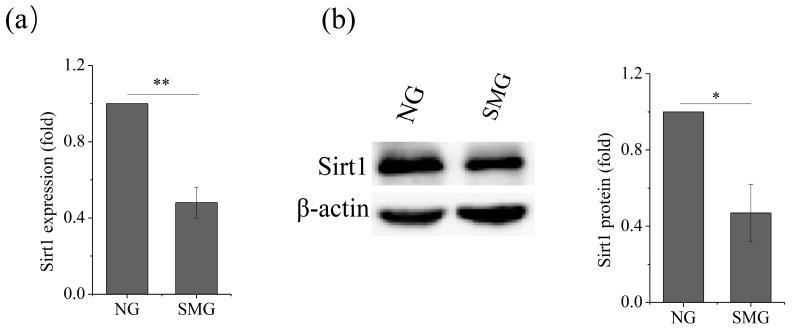
SMG inhibits the level of Sirt1 during osteogenesis of MSCs after exposure to SMG for 72 h. (**a**) Gene expression of Sirt1. (**b**) Protein levels of Sirt1. For each group, the values are the mean ± Scheme 0. * *p* < 0.05, ** *p* < 0.01. NG, normal gravity; SMG, simulated microgravity.

Studies showed that Sirt1 is a vital metabolic sensor and has a crucial role in osteogenic differentiation, we thus explored the effect of SMG on the expression of Sirt1. The results showed an evident downregulation of Sirt1 expression, both at the mRNA level (Figure 3a) and the protein level (Figure 3b), under the SMG conditions, as compared to that of NG, which indicated that the expression of Sirt1 was inhibited by SMG.

### 2.3. Upregulation of Sirt1 Enhanced OXPHOS

**Figure 4:**
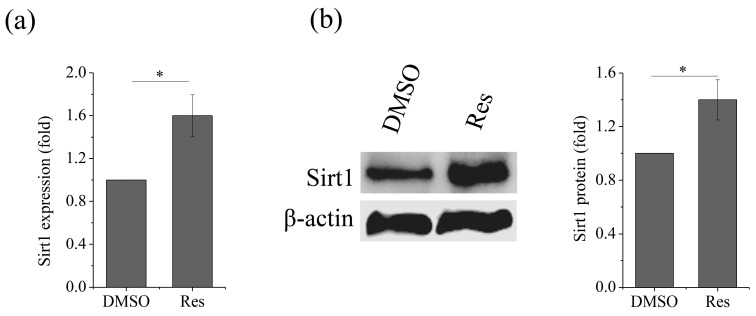
The expression of Sirt1 is upregulated after treatment with resveratrol. (**a**) Gene expression of Sirt1 after treatment with resveratrol. (**b**) Protein levels of Sirt1 after treatment with resveratrol. For each group, the values are the mean ± SEM from three representative independent experiments. Control—solvent control (DMSO). * *p* < 0.05. Res, resveratrol.

As Sirt1 is the key energy sensor, and the results above showed that both OXPHOS and Sirt1 decreased under SMG conditions, this drives us to further determine whether upregulation of Sirt1 could affect OXPHOS. First, we treated MSCs with 10 μM resveratrol, an activator of Sirt1, after which we observed that the Sirt1 expression significantly increased both at the mRNA (Figure S1a) and protein (Figure S2b) levels under NG conditions. We also observed that both the mRNA (Figure 4a) and protein (Figure 4b) levels of Sirt1 increased under SMG conditions after treatment with resveratrol.

**Figure 5:**
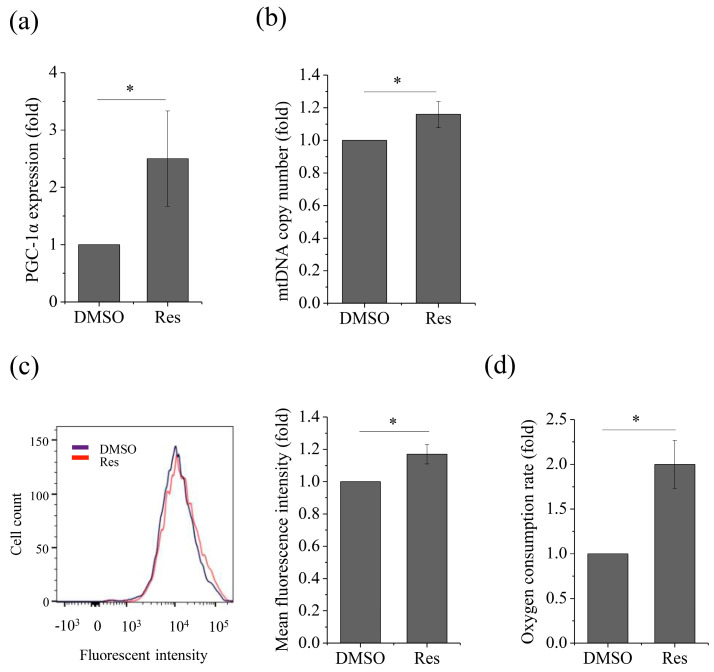
Upregulation of Sirt1 recovers OXPHOS of MSCs after exposure to SMG for 72 h with resveratrol treatment. (**a**) Gene expression of PGC-1α. The values are the mean ± SEM from four representative independent experiments. (**b**) Relative mtDNA copy number levels. The values are the mean ± SEM from four representative independent experiments. (**c**) Cells were stained with MitoTracker Green for mitochondrial mass and assessed by flow cytometry. The values are the mean ± SEM from three representative independent experiments. (**d**) Relative OCR levels. The values are the mean ± SEM from three representative independent experiments. Control—solvent control (DMSO). * *p* < 0.05. Res, resveratrol.

Then, we investigated the change of SMG-hampered OXPHOS, after activation of Sirt1. We first found that OXPHOS was increased by treating with 10 μM resveratrol under NG conditions, which was proved by increasing the expression of PGC-1α (Figure S2a), mtDNA copy number (Figure S2b), mitochondrial mass (Figure S3c), and OCR (Figure S4d). We next observed an increasing the expression of PGC-1α (Figure 5a), mtDNA copy number (Figure 5b), mitochondrial mass (Figure 5c), and OCR (Figure 5d) after resveratrol treatment under SMG conditions which led to ameliorated OXPHOS. These results suggest that upregulation of Sirt1 could restore SMG-hampered OXPHOS in MSCs, under SMG conditions.

### 2.4. Improving OXPHOS via Sirt1 Rescued Osteogenic Differentiation of MSCs

**Figure 6:**
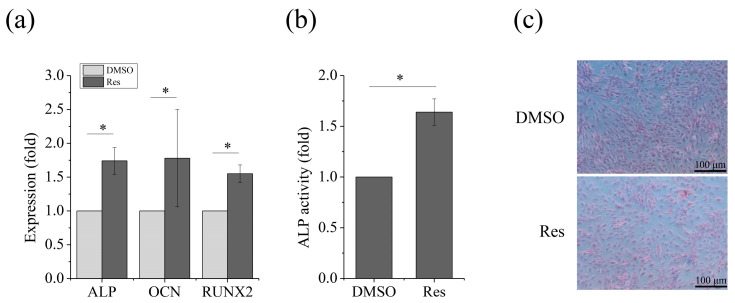
Enhancing OPXHOS via Sirt1 recovers osteogenesis of MSCs after exposure to SMG with resveratrol treatment. (**a**) Gene expression of ALP, OCN, and RUNX2 after 72 h. (**b**) Relative ALP activity after 72 h. (**c**) Representative images of ALP staining after 7 day. For each group, the values are the mean ± SEM from three representative independent experiments. Control—solvent control (DMSO). * *p* < 0.05. Res, resveratrol. Scale bar: 100 μm.

To evaluate whether MSC osteogenesis could be rescued under SMG conditions when OXPHOS was enhanced, we investigated the expression of osteogenic markers with resveratrol treatment. As expected, increase in the expression of ALP, OCN and RUNX2 (Figure 6a) and ALP activity (Figure 6b) as well as an increase for positive ALP staining of cells (Figure 6c) were observed. These results demonstrated that improving OXPHOS by upregulation of Sirt1 using resveratrol could lead to recovery of osteogenic differentiation of MSCs under SMG conditions.

## 3. Discussion

The data in these study indicate that activating OXPHOS via Sirt1 could reverse the inhibition of SMG on osteogenic differentiation of MSCs. To our knowledge, this is the first study demonstrating that SMG hindered osteogenic differentiation of MSCs by affecting OXPHOS. Moreover, our findings propose a new strategy for repairing bone loss under SMG conditions by enhancing OXPHOS via Sirt1, which is meaningful for explaining and treating bone loss under microgravity conditions.

In recent years, a growing number of studies found that stem cell differentiation is related to energy metabolism. It was reported that MSCs prefer glycolysis for energy supply [17], but when MSCs commit to differentiation, much more energy is required for differentiation, which is consistent with the activation of OXPHOS [18]. Studies confirmed that metabolism can shift to OXPHOS from glycolysis during osteogenesis [5,18] and that OXPHOS is necessary for osteogenesis [19]. Morabito et al. showed that extracellular glucose uptake and lactate level of the MC3T3-E1 cells, a widely used osteoblast-like phenotype, was reduced by microgravity, meanwhile, they observed a reduction of mitochondrial membrane potential under microgravity conditions, indicating that microgravity could damage mitochondria [20]. Considering these results mentioned before, we hypothesize that metabolism shift in MSCs might also occur under microgravity conditions. In the present study, our results indicated that SMG could inhibit OXPHOS during osteogenic differentiation. Hence, we proposed that OXPHOS, which was repressed by SMG, led to the inhibition of osteogenic differentiation of MSCs under SMG conditions, which was confirmed by the results that the osteogenic potential of MSCs was reduced after inhibiting OXPHOS with its inhibitor [7]. Moreover, we found that enhancing OXPHOS rescued osteogenesis of MSCs under SMG conditions. These findings suggest that SMG inhibits osteogenesis, possibly through OXPHOS.

It was shown that mitochondria are dynamic organelles undergoing biogenesis, fission, fusion, mitophagy, and motility, and when mitochondrial fission is promoted, mitochondrial fusion and OXPHOS should be repressed [21]. Moreover, the mtDNA increases when the balance between mitochondrial fission and fusion is broken [22]. In this context, the increased mtDNA copy number under SMG conditions in this study could possibly be explained by the imbalance between mitochondrial fission and fusion.

Reports showed that Sirt1 is the key energy sensor and can mediate mitochondrial biogenesis by regulating PGC-1α and its downstream genes, NFR1, NRF2, and TFAM, resulting in the enhancement of OXPHOS [23,24]. Additionally, deletion of SIRT1 could reduce the mRNA levels of all mitochondrially encoded OXPHOS genes [25], which suggests Sirt1 is the crucial factor for regulating oxidative metabolism. Moreover, evidence from several studies showed that Sirt1 has an important role in regulating osteogenesis [10,11,12,13]. In this study, we tested the hypothesis that Sirt1 could modulate osteogenesis of MSCs under microgravity conditions. We found that the expression of both Sirt1 and PGC-1α decreased under SMG conditions during osteogenesis of MSCs, which suggested that the Sirt1/PGC-1α signaling involved in energy metabolism could affect osteogenesis under SMG conditions.

Osteoporosis can be caused by oxidative stress. Mechanical stretching was shown to attenuate intracellular reactive oxygen species by inducing antioxidant responses via activating Sirt1, and improving osteogenesis of MSCs [26]. Microgravity can lead to bone loss, resulting in osteoporosis. Our results showed that SMG repressed the expression of Sirt1 during osteogenesis, suggesting that oxidative stress was enhanced. SMG could activate oxidative stress by repressing Sirt1, followed by antioxidant enzymes, resulting in inhibition of osteogenic differentiation of MSCs.

To observe whether the upregulation of Sirt1 could affect OXPHOS during osteogenesis under SMG conditions, we treated MSCs in SMG with resveratrol, an activator of Sirt1, which was correlated with PGC-1α and mitochondrial biogenesis [16,27,28]. As expected, OXPHOS was activated after treatment with resveratrol under SMG conditions, accompanied by the recovery of osteogenesis. Our data demonstrated that SMG could affect OXPHOS via Sirt1, then lead to the inhibition of osteogenesis in MSCs.

**Figure 7:**
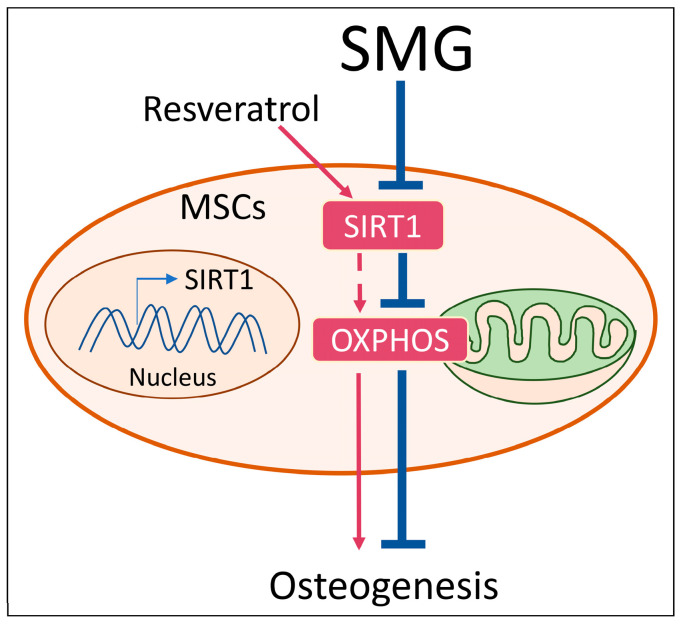
Proposed model by which SMG inhibits OXPHOS and thus hampers osteogenesis of MSCs. After exposure to SMG, Sirt1 was downregulated and OXPHOS was reduced, followed by the suppression of osteogenesis. Upon resveratrol treatment, Sirt1 was upregulated and OXPHOS was enhanced, and thus osteogenesis was recovered under SMG conditions. Collectively, SMG could induce a decrease in OXPHOS to inhibit osteogenesis of MSCs.

In summary, SMG can inhibit OXPHOS to impair osteogenic differentiation of MSCs, which was possibly due to the decreased expression of Sirt1. Activation of OXPHOS induced by resveratrol rescued osteogenic differentiation of MSCs, primarily through Sirt1. Thus, our results suggest that OXPHOS is an important factor for retaining osteogenic differentiation of MSCs in SMG conditions and that both OXPHOS and Sirt1 are potential therapeutic targets for the SMG-inhibited osteogenic differentiation of MSCs. A schematic diagram summarizing the proposed model of the reduction of the osteogenic potential of MSCs induced by SMG is shown in Figure 7, which indicates that energy metabolism is involved in osteogenesis under SMG conditions. The findings in this study could provide new insights into better understanding OXPHOS, osteogenic differentiation of MSCs, and bone loss under microgravity conditions.

## 4. Materials and Methods

### 4.1. Cell Isolation and Treatments

All animal experimental procedures were performed in accordance with the ethical standards and national and international standards, and were approved by Chongqing Science and Technology Commission, Chongqing, China. All male Sprague-Dawley (SD) rats used in this study weighed approximately 150 g and were provided by the Chongqing Medical University. The MSCs were isolated from the SD rats through the method of whole bone marrow adhesion. Briefly, the femurs and tibia from the SD rats were separated, and the bone marrow was flushed with a syringe under sterile conditions. The MSCs were then enriched and cultured in low-glucose Dulbecco’s modified Eagle’s medium (DMEM, Gibco, Grand Island, CA, USA), with 10% fetal bovine serum (FBS, Gibco, Grand Island, CA, USA), penicillin (100 U/mL), and streptomycin (100 μg/mL), in an incubator with 5% CO_2_ at 37 °C. In this study, all cells used were between passage 2 and passage 4. For osteogenic differentiation, when the cells reached the proper confluency conditions, the culture medium was changed to the differentiation medium containing 10% FBS, 100 nM dexamethasone (Sigma, Saint Louis, MO, USA), 50 mM β-glycerol phosphate (Sigma, Saint Louis, MO, USA), and 50 μg/mL ascorbic acid (Sigma, Saint Louis, MO, USA). In this study, all assays were performed under 21–22% oxygen.

### 4.2. Production of Simulated Gravity Conditions

A two-dimensional clinostat device was utilized to simulate microgravity, which was constructed by the National Microgravity Laboratory, Institute of Mechanics, Chinese Academy of Sciences, China. Clinostats can stimulate effects similar to microgravity through continuous rotation, to constantly change the cell orientation with regards to gravity [29,30]. The apparent gravity on cells could be reduced by the clinostat in this experiment to approximately 10^−3^ g, compared with normal gravity (1 g), when the clinostat rotated at 10 rpm. The photograph and principle of the clinostat to stimulate microgravity is shown in Figure 1. The cells were attached on the cell culture slide with constant changes in orientation, followed by changes of the gravity vector. The gravity vector was nulled after rotating one circle, which meant that the average gravity vector equaled 0 g. As previously described, the MSCs were seeded into each chamber at a total of 3 × 105 cells, ensuring that no air bubbles existed in the chamber to prevent fluid shear stress. After the cells were attached to the slide for 24 h and reached a confluency of 80%, the chambers were fixed onto the clinostat, and the effect of simulated microgravity was achieved by rotation around the horizontal axis at 10 rpm for 72 h, in an incubator with 5% CO_2_ at 37 °C. For normal gravity (NG) controls, static cells were cultured in the same chamber under the same conditions but without fixing onto the clinostat.

### 4.3. Alkaline Phosphatase Activity Assay

For the quantitative assessment of alkaline phosphatase (ALP) activity, the MSCs were first incubated for 1 h at room temperature, with 500 μL of 0.1% Triton X-100 (Sigma-Aldrich, Saint Louis, MO, USA) for cell lysis, and the cell supernatant, was collected in a 96-well plate. Next, buffer from the Alkaline Phosphatase Assay Kit (Wanleibio, Shenyang, China) was subsequently added according to the manufacturer’s instructions, and incubated for 15 min at 37 °C. The absorbance was measured at a wavelength of 490 nm, using a microplate reader. These results were normalized to the total intracellular protein content determined by the bicinchoninic acid (BCA) Protein Assay Kit (Beyotime, Shanghai, China).

### 4.4. Alkaline Phosphatase Staining

For ALP staining assay, the MSCs were first fixed with 500 μL of 4% formaldehyde (Solarbio, Beijing, China) for 15 min at room temperature. Working solution from the Alkaline Phosphatase Staining Assay Kit (Solarbio, Beijing, China) was subsequently added according to the manufacturer’s instructions, and incubated for 20 min at room temperature in the dark. Next, the staining solution was added and incubated for 5 min at room temperature. Images were then obtained using a light microscope (Leica, Wetzlar, Germany).

### 4.5. Measurement of the Oxygen Consumption Rate

The oxygen consumption rate (OCR) was measured using the Oxygen Consumption Rate Assay Kit (Cayman, Ann Arbor, MI, USA), according to the manufacturer’s instructions. In brief, the MSCs were plated on black, clear-bottom 96-well tissue culture-treated plates for 24 h before the experiment, at a density of 8000 cells/well in 200 μL. The media was replaced with 160 μL fresh culture medium and 10 μL phosphorescent oxygen probe solution, immediately before the experiment. Each well was gently overlaid with 100 μL of mineral oil. The fluorescence signal was then obtained on a plate reader at the excitation and emission wavelengths of 380 and 650 nm, respectively, at 37 °C for 2 h. The OCR was normalized to the total cell number.

### 4.6. mtDNA Copy Number Analysis

For mtDNA copy number analysis, total DNA was isolated using a DNA extraction kit (Magen, Guangzhou, China), according to the manufacturer’s instructions. The total extracted DNA concentration was determined using a Nanodrop 2000 spectrophotometer (Thermo Fisher Scientific, Waltham, MA, USA). The mtDNA was assessed by quantitative polymerase chain reaction, to determine the copy number of the mtDNA-encoded gene for NADH dehydrogenase 1 (ND1) subunit, with nuclear-encoded β-actin as the internal control. The sequences of the PCR primer pairs for amplification were as follows—the ND1 fragment was amplified by primer pair 5′-GGAGTAATCCAGGTCGGT-3′ and 5′-TGGGTACAATGAGGAGTAGG-3′; the β-actin fragment was amplified by primer pair 5′-CATGTGCAAGGCCGGCTTC-3′ and 5′-CTGGGTCATCTTCTCGCGGT-3′.

### 4.7. Mitochondrial Staining Assay

Mitochondrial mass was estimated by MitoTracker Green stained mitochondria [31]. In brief, the cells were stained with MitoTracker Green (Beyotime, Shanghai, China) according to the manufacturer’s instructions. The MSCs were incubated with MitoTracker Green mixed into cell culture medium at 37 °C, in the dark for 30 min. The cells were then washed in PBS, detached by trypsin, and resuspended in PBS with 2% FBS. The fluorescent intensity of mitochondria was obtained by flow cytometry (BD Biosciences, Franklin Lakes, NJ, USA).

### 4.8. Quantitative Real-Time Polymerase Chain Reaction (qRT-PCR)

Total cellular RNA was isolated using an RNA extraction kit (Bioteke, Beijing, China), according to the manufacturer’s instructions. The total extracted RNA concentration was determined using a Nanodrop 2000 spectrophotometer (Thermo Fisher Scientific, Waltham, MA, USA). A reverse transcription reaction was then performed with 1 μg of the extracted RNA reversely transcribed to cDNA, using the PrimeScript™ RT reagent kit with gDNA eraser (TaKaRa, Kusatsu, Japan). Next, PCR was performed using a CFX96™ real-time PCR detection system (Bio-Rad CFX Manager system, Hercules, CA, USA) with SYBR Premix Ex Taq (Bioteke, Beijing, China), according to the manufacturer’s instructions. The PCR reaction conditions used were 30 s at 95 °C, followed by 40 cycles at 95 °C for 5 s and 40 s at 60 °C. The primer sequences are available in Table 1. The expression of the targeted gene was normalized to the expression of β-actin.

### 4.9. Western Blot

The total protein of the MSCs was collected on ice using SDS–PAGE loading buffer (Solarbio, Beijing, China) supplemented with a protease inhibitor cocktail or phosphatase inhibitor cocktail (Roche, Branchburg, NJ, USA), followed by boiling for 5 min at 100 °C, for denaturation. The protein aliquots were separated by 10% *v*/*v* SDS–PAGE and electroblotted onto PVDF membranes (Millipore, Billerica, MA, USA). The membranes were blocked with tris-buffered saline (TBS) containing 0.1% tween 20 (TBST) and 5% *v*/*v* non-fat milk, for 1 h, at room temperature. Antibodies against Sirt1 (Cell Signaling Technology, Danvers, MA, USA), and β-actin (ZSGB-BIO, Beijing, China) were used according to the manufacturers’ instructions. The incubation was performed overnight at 4 °C. The membrane was then washed three times with TBST and incubated with horseradish peroxidase-conjugated antibody (ZSGB-BIO, Beijing, China), for 1 h at room temperature. The target proteins’ expression was visualized using an enhanced electrogenerated chemiluminescence (ECL) system (VersaDoc, Bio-Rad, Hercules, CA, USA). The expression of Sirt1 was normalized to the expression of β-actin.

### 4.10. Statistical Analysis

All data were normalized to the control group and expressed as the mean ± standard error of mean (S.E.M.). Each assay was performed a minimum of three times. The significant differences were determined using the two-tailed Student’s *t*-test for comparison between the two groups. When *p* < 0.05, the data between two groups was considered to have statistically significant differences.

## Figures and Tables

**Table 1:** Primers used for quantitative real-time polymerase chain reaction.

Genes	Forward Primer Sequence (5′-3′)	Reverse Primer Sequence (5′-3′)
ALP	TCGGACCCTGCCTTACCA	TGTCTCCTCGCCCGTGTT
OCN	AGCTCAACCCCAATTGTGAC	AGCTGTGCCGTCCATACTTT
RUNX2	CAGACCAGCAGCACTCCATA	CAGCGTCAACACCATCATTC
PGC-1α	TGACCACAAACGATGACCCTC	GACTGCGGTTGTGTATGGGAC
Sirt1	GGAACCTCTGCCTCATCTA	CATACTCGCCACCTAACCT
β-actin	ACCGTCAGGTCACTATCG	GGCATAGAGGTCTTTACGGATG

## Supplementary Materials

Supplementary materials can be found at https://www.mdpi.com/1422-0067/21/24/9747/s1.

## Abbreviations

SMG	Simulated Microgravity
MSCs	Mesenchymal Stem Cells
OXPHOS	Oxidative Phosphorylation
PGC-1α	Peroxisome Proliferator Activated Receptor γ Coactivator 1α
mtDNA	Mitochondrial DNA
OCR	Oxygen Consumption Rate
Sirt1	Sirtuin 1
ALP	Alkaline Phosphatase
OCN	Osteocalcin
RUNX2	RUNX Family Transcription Factor 2
Res	Resveratrol
NG	Normal Gravity
