# Supplementary material for: Simulated Microgravity Suppresses Osteogenic Differentiation of Mesenchymal Stem Cells by Inhibiting Oxidative Phosphorylation
Source: Int J Mol Sci. 2020 Dec 21;21(24):9747. doi: 10.3390/ijms21249747 (PMC7767150; doi:10.3390/ijms21249747)
Supplement: Supplementary file 1 [file ijms-21-09747-s001.pdf]

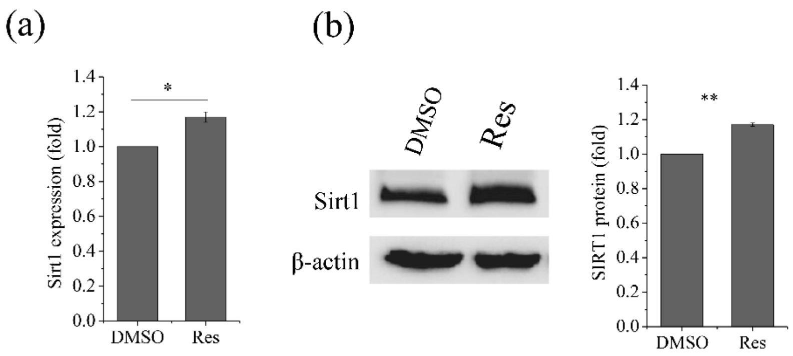

Figure S1. The expression of Sirt1 is upregulated after treatment with resveratrol under normal gravity conditions. (**a**) Gene expression of Sirt1 after treatment with resveratrol; (**b**) Protein levels of Sirt1 after treatment with resveratrol. For each group, the values are the mean ± SEM from three representative independent experiments. Control: solvent control (DMSO). *$p < 0.05$. Res, resveratrol.

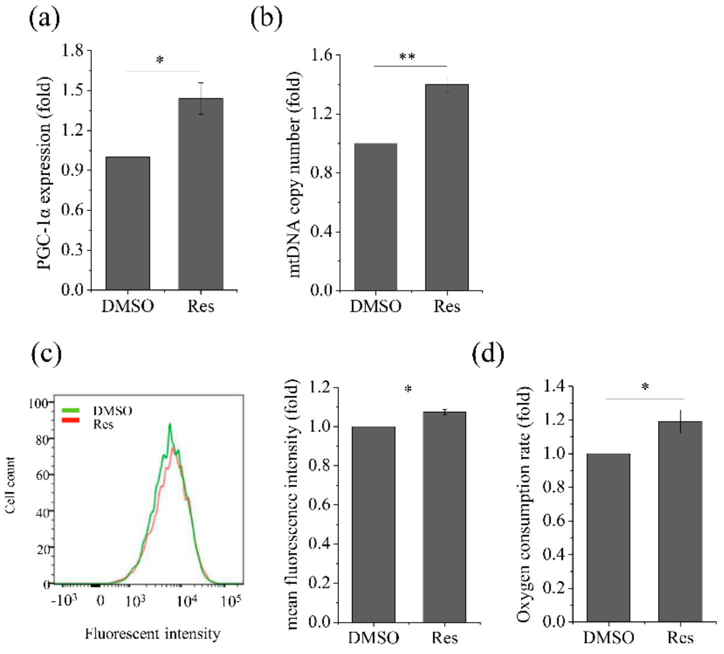

Figure S2. Upregulation of Sirt1 recovers OXPHOS of MSCs for 72h with resveratrol treatment under normal gravity conditions. (**a**) Gene expression of PGC-1α. (**b**) Relative mtDNA copy number levels. (**c**) Cells were stained with MitoTracker Green for mitochondrial mass and assessed by flow cytometry. (**d**) Relative OCR levels. The values are the mean ± SEM from three representative independent experiments. Control: solvent control (DMSO). *$p < 0.05$. Res, resveratrol.
